# Development and Validation of the Adolescent and Children in Risk of Abuse and Maltreatment Protective Factors Scale (ACRAM-PFS)

**DOI:** 10.1007/s10560-022-00908-7

**Published:** 2023-01-03

**Authors:** Adrián García-Mollá, Ángela Carbonell, José Javier Navarro-Pérez, José M. Tomás

**Affiliations:** 1 Department of Methodology for the Behavioral Sciences, University of Valencia, Valencia, Spain; 2 Department of Social Work and Social Services, University of Valencia, Tarongers Avenue, 4B, 46021 Valencia, Spain

**Keywords:** Child maltreatment, Child welfare, Protective factors, Assessment

## Abstract

**Background:**

Child maltreatment is a significant global problem concerning over 25% of children around the world. Traditionally, the assessment of children’s welfare was characterized by the creation of instruments and models from the deficit-based theoretical framework.

**Purpose:**

This study aims to develop an instrument to measure protective factors (the Adolescent and Children Risk of Abuse and Maltreatment Protective Factors Scale, ACRAM-PFS) and gather evidence on its psychometric properties. ACRAM-PFS is an 18-items scale for the assessment of protective factors of child maltreatment developed from the socioecological framework.

**Method:**

Structural validity, reliability and convergent-related validity were studied for this measure in a sample of 616 children and adolescents, with age ranging from 0 to 18 years old (M = 12.14; SD = 5.22). Cases were informed by 286 child welfare workers. The sample was split in two subsamples, one to perform an Exploratory Factor Analysis (EFA) and the second to perform a Confirmatory Factor Analysis (CFA).

**Results:**

The CFA corroborate the three-factor structure that includes the children and adolescents’ resources, the family/caregiver’s resources and the community resources (χ^2^ = 278.005, df = 132, *p* < .001, CFI = 0.955, SRMR = .084, RMSEA = .061, [90% CI: .051-.071]). Results of convergent-related validity indicated significant correlation with CTQ-SF and protective factors dimension of C-CAPS.

**Discussion:**

The results support that ACRAM-PFS is a rigorous measure for assessing protective factors for child maltreatment. The scale can serve as a key tool for designing strengths-based intervention strategies tailored to the actual needs of children and adolescents. The present study provides the implications for the development of protective factor scales in the field of child welfare.

Child protection is one of the vital areas of professional social work, and diagnosis in child and adolescent maltreatment cases may have important consequences for children, adolescents, and their families. As defined by WHO (2020a), maltreatment can be expressed in different ways like physical or emotional abuse, sexual abuse, neglect and commercial or other exploitation, which jeopardizes children's development and gives rise to multiple harmful consequences in health and social functioning. Moreover, this is an issue that entails medical resource costs as demonstrated by Florence et al. (2013) and high financial costs in countries like United Kingdom (Conti et al., 2021), France (Prigent et al., 2021) or United States (Letourneau et al., 2018; Peterson et al., 2018). Child maltreatment should therefore be considered a major global public health problem.

The exposure to adverse situations during childhood and adolescence generates stressful developmental settings promoting adaptative problems on various domains like academic functioning (Fisher & Widom, 2021), psychosocial functioning (Alink et al., 2012), mental health (Cohen et al., 2019; Danese & Widom, 2020; Humphreys et al., 2020; VanMeter et al., 2021) or aggressive behavior (Augusti et al., 2018). Additionally, long term consequences of child maltreatment have also been identified among adults who suffered abuse or neglect during childhood (Mehta et al., 2021). In this line, it is widely known that youth that experienced maltreatment are more likely to use substances (Burlaka et al., 2019; Herrenkohl et al., 2013), to develop mental illness (McKay et al., 2021), and to have economic difficulties during adulthood (Henry et al., 2018), leading them to a social exclusion situation.

Traditionally, explanatory models on child welfare settings are based on socioecological models which postulates that child maltreatment is a consequence of complex association of variables. Belsky (1980, 1993) proposed an ecological model to study the etiology of child maltreatment grouping the causal factors into different nested systems. Based on Bronfenbrenner’s model (1979), Belsky proposed three systems: the individual, the proximate system, and the distant system, interacting with each other and having an impact on the development of the individual. More proximate influences and their immediate care environment will generally have a greater impact on their development, while more distant influences will have indirect or less severe effects.

These factors include risks associated with parental or family relationships and behaviors, biological and psychological characteristics of the child or adolescent, and factors associated with the environment and community (Belsky, 1980, 1993; Lakhdir et al., 2021). In a complementary way, the model includes protective factors for child maltreatment, which are defined as conditions or circumstances capable of favoring the children or adolescent’s development, such as social support or family resources, and reducing or offsetting the impact of other high-risk variables (Conrad-Hiebner et al., 2019; Ridings et al., 2017). Protective factors should not be conceptualized merely as the absence of risks but should be treated as positive conditions that may moderate the negative influences of risks experienced by children (Shen & Hannum, 2022). As Austin et al. (2020) pointed out, this combination allows for a holistic analysis of the causes and consequences of child maltreatment.

## Detecting and Measuring Abuse: From Risks to Promoting Strengths

Preventive actions of child maltreatment have typically focused on the identification and the study of risk factors that are causing or promoting this maltreatment. The presence of risk factors is understood as higher probability to suffering child maltreatment or to have worse prognosis, while protective factors reduce that probability and are related to positive outcomes despite the exposure to adverse situations (Masten & Garmezy, 1985). However, it is known that risk factors are less sensitive to the short- or medium-term effects of intervention programs given the structural nature and the stability of that variables. Therefore, protective factors may be better indicators than risk factors of the effectiveness of intervention programs (Ross & Vandivere, 2009).

Protective factors have shown to decrease the probability to develop a distress disorder after being exposed to adverse situations (Racine et al., 2020) as well as the likelihood of child-to-parent violence (Beckmann et al., 2017). In addition, intervention programs that promote self-compassion and inclusion on the school setting result in a reduction of suicidal ideations in victims (Zhang et al., 2021a, 2021b), and the presence of inherent protective factors also reduces the risk of suicide in children (Janiri et al., 2020). In cases of institutionally abused children, protective factors also moderate the prevalence of negative psychosocial outcomes (Carr et al., 2019). Due to these benefits, from 2000 onwards, child maltreatment prevention programs aimed at reducing family risks shifted their focus to promoting family strengths (Browne, 2014).

In this vein, positive psychology emphasizes positive influences on protective factors and personal, family and contextual strengths on children or adolescent’s well-being (Miller-Perrin & Mancuso, 2015; Wu et al., 2018). Thus, conclusions drawn from research carried out by Ginsburg (1986) point out that strengths-based theories allow for the development and creation of assessment scales and treatments aimed at identifying and promoting positive traits. In the last years, research on intervention through positive psychology shows that it is a more effective approach than deficit-focused models, as it provides evidence of progress and growth in positive aspects (Owens & Waters, 2020). These theories evolved from nursing to education and community social work and were based on addressing needs and correcting them. But also by identifying risks in order to plan processes and fill gaps. Without resorting to other strategies, based on the qualities (Crescenza et al., 2021) or the positive disposition of the individual, which could strengthen both the individual and collective sphere, and thus generate new potentialities. Despite these advantages, the assessment of child well-being still focuses on identifying gaps and risks in order to address and modify them (Yoo et al., 2022), or on identifying protective factors as mere absences of risks rather than as targets and possibilities for intervention (Liel et al., 2022; Soderstrom et al., 2020).

Therefore, according to LeBuffe and Shapiro (2004) and Counts et al. (2010), measures in the child maltreatment field are still created following the deficit-focused theoretical model, while scales directly assessing protective factors are scarce in the scientific literature. The systematic review by Georgieva et al. (2022) concluded that existing scales had a notable lack of information and limited scientific evidence of reliability and validity, being most of these instruments self-informed. According to Frazier (2018) or Kim et al. (2022) these instruments can be subjective in assessing one’s own abilities, especially in children and adolescents who have experienced adversity and may have an altered self-concept. The relevance of protective factors in relation to prevention and evaluation of intervention programs generates the need to create specific scales with good psychometric properties designed to be filled out *externally* by qualified professionals (Counts et al., 2010; Navarro-Pérez et al. 2022a). In addition, Dimitrova and Wiium (2021) recently promoted the development of instruments within the framework of public policies that are accompanied by qualitative sections to develop lines of intervention based on the strengths of the child, their family or the natural environment in which they socialise and that promote resilient actions. In this vein, Domhardt et al. (2015) highlighted that negative consequences following child maltreatment can be prevented or moderated if protective factors are provided early, encouraging research on this issue and warning that delaying the detection of protective factors affects children's future resilience.

Recent studies highlighted the role of socioecological models with the aim to gather as much information as possible from the complex situations where child maltreatment occurs (Austin et al., 2020). Despite the reduced number of comprehensive measures created to assess the totality of protective factors (Navarro-Pérez et al. 2022a; Sprague-Jones, 2019), most of them are designed specifically to assess one of the multiple dimensions of protective factors like, for example, child or family traits (Counts et al., 2010; García-Grau et al., 2018), resilience (Daigneault et al., 2013; Llistosella et al., 2019), or school engagement (Cage et al., 2019). As defined by Clauss-Ehlers (2008), the development of resilience is influenced by contextual factors, and not only characterized by children intrinsical traits. This fact therefore justifies the need to pay attention to the different factors (personal, family and contextual) that favor positive outcomes and to leave the deficit-based approach behind (Cui et al., 2020).

## Current Study

The effective detection and assessment of not only risks but also protective factors in childhood and adolescence makes it possible to face a subsequent scenario with fewer risks and to transform the existing risks into intervention objectives from the welfare services. ACRAM (Adolescent and Children Risk of Abuse and Maltreatment) arises as a response to the need and demand of professionals, experts, and researchers (WHO, 2020b; Unicef, 1989) to have a common, valid and reliable tool that makes it possible to draw up standardized and objective diagnoses, to guide professional decision-making, and to assess situations of risk and lack of protection of children and adolescents. ACRAM is a battery of questionnaires covering parental and caregiver risk factors (ACRAM-PS), community-related factors (ACRAM-CFS), protective factors (ACRAM-PFS) and other factors related to the complexities associated with unaccompanied asylum seeker children (ACRAM-US).

There is a lack of studies in the literature dealing with measuring or assessing protective factors of child maltreatment. Additionally, the general lack of psychometric evidence in the scales measuring different dimensions of protection from child maltreatment, gives rise to the need to develop a protective factors inventory for the adequate identification of these variables (Navarro-Pérez et al. 2022a; Meng et al., 2018), and offer evidence on its psychometric behavior. In order to address this last objective, we examined the structural validity and internal consistency of this new scale called Adolescent and Children Risk of Abuse and Maltreatment Protective Factors Scale (ACRAM-PFS), developed on the basis of the socio-ecological model proposed by Belsky (1980, 1993). To our knowledge, this is the first hetero-administered protective factor scale that includes indicators relating to the three systems and the resulting dimensions are referred to as: children/adolescents’ resources, family/caregiver’s resources, and community resources. This instrument will be useful to carry out more comprehensive, accurate and effective interventions on children and adolescents involved in this situation.

## Method

The sample employed in this study comes from the first wave of DAP 360º, a longitudinal study carried out in the Valencian Community (Spain) aimed to develop an instrument for the comprehensive detection and assessment of child maltreatment. The result of DAP 360° project is the ACRAM, a battery of child maltreatment risk and protective indicators designed according to the needs of child welfare workers and submitted to a content validity process (Carbonell et al., 2022; Navarro et al., 2022b).

### Sample and Procedure

The sample was gathered by convenience sampling. We contacted and trained child welfare professionals from different child protective services (CPS) to fill out the questionnaire to inform about cases they were working on using an online survey.

A total of 286 child welfare workers informed about cases of 635 children and adolescents attended by different CPS from the Valencian community (Spain). Given the absence of responses on all items of ACRAM-PFS, 19 participants (2.99%) were excluded from the statistical analysis. Therefore, the final sample consisted of the assessments of 616 individuals; their age ranged from 0 to 18 years old (M = 12.14; SD = 5.22). The 42.5% were female, 56.8% male, and 0.6% declared non-binary gender. Data comprised 36 nationalities being the most common Spanish (77.3%) and Moroccan (7.1%). From the whole sample, a total of 52.9% were in a low or moderate risk situation, while 46.6% were at high risk and had been removed from their homes. Table 1 includes descriptive statistics about the final sample.

**Table 1:** Sample’s descriptive statistics (N = 616)

Variable	*n* (%)	Mean (SD; range)
Gender		
Female	262 (42.5)	
Female	350 (56.8)	
Non-binary	4 (0.6)	
Age		12.14 (5.22; 0–18)
Nationality		
Spain	476 (77.3)	
Morocco	44 (7.1)	
Romania	13 (2.1)	
Colombia	13 (2.1)	
Algeria	12 (1.9)	
Child protective measure		
Low and moderate risk	326 (52.9)	
High risk	287 (46.6)	

The sample was randomly split in two subsamples. The first subsample comprised 301 participants employed to perform an Exploratory Factor Analysis (EFA), while the second one, composed of 315 cases, was used to perform a Confirmatory Factor analysis (CFA).

This research complied with APA’s ethical standards, and it was approved by the Ethical Commission of the Valencian Government (CSV:HYH5NVSA-Y85ZSB11-RML6ZCYX). The children’s data were all anonymous, and all professionals signed informed consents.

### Instruments

We employed three scales in this study: the one under development and validation (ACRAM-PFS), and two more with validation purposes (the Childhood Trauma Questionnaire—Short Form and the Cleveland Child Abuse Potential Scale).

The ACRAM (Carbonell et al., 2022; Navarro et al., 2022b). It is a comprehensive instrument for the detection and assessment of child maltreatment including 97 risk or protective indicators divided into three general sections: (1) Risk factors associated to parental/caregiver behavior (2) Risk factors associated to the environment, and (3) Protective factors. In this study, Section 3 formed by the Adolescents and Children Risk of Abuse and Maltreatment Protective Factors Scale (ACRAM-PFS) is examined. ACRAM-PFS is a protective indicators inventory designed to be filled in by child welfare professionals. During the theoretical development of the scale, it was pretended to include protective indicators related to the children or the adolescent, the family or caregiver and the community characteristics. The scale was formed by 18 items rated on a three-point response scale: 0 (there is clear evidence it does not occur), 1 (there are signs it might occur, but it cannot be confirmed) and 2 (there is clear evidence it does occur).

In order to develop the scale of protective factors, firstly, a systematic search of national (Spanish) and international scientific literature was carried out in order to find instruments for detecting and assessing protection dimensions. Secondly, a pre-diagnosis study was carried out. 144 professionals (mostly educators and social workers) from 20 primary healthcare social services teams from the child and adolescent program in different towns and cities in The Valencian Community participated in the content validity assessment with mixed research techniques. These mixed techniques were, on the one hand, interviews and focus groups, and on the other, the quantitative analysis of data relating to the tool’s relevance and the understanding of its content obtained from questionnaires in order to guarantee inter-rater agreement. Thirdly, a pilot version of the scale was developed and 15 focus groups further refine the content validity of the scale. These focus groups were formed of 80 professionals from different areas of child and adolescent care services.

The Childhood Trauma Questionnaire—Short Form (CTQ-SF; Bernstein et al., 2003), is a 28-item scale of retrospective child abuse and neglect developed from the original 70 items’ version (Bernstein et al., 1994). We employed an actuarial version of CTQ-SF translated to Spanish that assesses different dimensions of maltreatment: emotional abuse, physical abuse, sexual abuse, emotional neglect, and physical neglect. The scale presented adequate reliability in this sample with α = 0.92 for emotional abuse, α = 0.75 for physical abuse, α = 0.95 for sexual abuse, α = 0.73 for physical neglect and α = 0.93 for emotional neglect. The response scale ranged from 1 (never true) to 5 (very often true).

The Cleveland Child Abuse Potential Scale (C-CAPS; Ezzo & Young, 2012) is an actuarial risk assessment scale of 26 items based on factual variables that do not require interpretation by the interviewer. In this study we were only interested in its protective factors dimension. Items comprising this dimension were translated to Spanish and showed adequate reliability (α = 0.81). Answers were rated on a three-point scale: 0 (there is clear evidence it does not occur), 1 (there is partial evidence it occurs) and 2 (there is clear evidence it occurs).

### Statistical Analyses

We analyzed several psychometric properties in current research. Specifically, we tested for factorial validity, internal consistency (reliability), and convergent validity. Factorial validity refers to the clustering of correlations of responses to the items in the scales. Factor analysis is a statistical modeling technique that aims to explain the common variability among a set of manifest variables or indicators with a reduced set of variables known as factors or dimensions, and can be used to test for factorial validity. This can be done with either exploratory or confirmatory factor analyses, depending on how articulated is the theory about what the scale is measuring. Reliability refers to the consistency of the results from a measurement instrument. For example, we may consider test–retest reliability or estimates of internal consistency, and if low values are present the scale has a large amount of measurement error. Finally, convergent validity refers to how closely a scale (instrument) is related to other scales (instruments) that measure the same constructs or other constructs closely related. Here, we understand construct as a behaviour, attitude, or concept that is not directly observable.

Different analyses were performed to assess the factorial structure of ACRAM-PFS. Firstly, an exploratory factorial analysis (EFA) was performed in the first random split of the sample in order to examine the factor structure of the questionnaire. Secondly, to validate the results of the EFA, a confirmatory factor analysis (CFA) was performed in the second random split of the sample to validate the best performing EFA structure found in the previous analyses. The estimation method employed, both for the EFA and CFA models was WLSMV, due to is largely known to perform well when using categorical non-normal data (Finney & DiStefano, 2013).

In order to analyze model’s fit, several statistics and indexes were used. Specifically, we employed the chi-square statistic (χ^2^), the Comparative Fit Index (CFI), the Standardized Root Mean Square Residual (SRMR), and the Root Mean Squared Error of Approximation (RMSEA). Acceptable model fit is considered with values equal or greater than 0.90, and RMSEA and SRMR values equal or lower than 0.08 (Hu & Bentler, 1999). Main analyses were performed using Mplus 8.7 (Muthén & Muthén, 2017), while descriptive statistics and zero-order correlations were calculated in SPSS 26. Inter-item correlations, also calculated in SPSS 26, are offered with descriptive purposes. Additionally, internal consistency of the ACRAM-PFS dimensions was estimated using the McDonald’s Omega in order to overcome some limitations of Cronbach’s alpha (Hancock & An, 2018).

In sum, the hypotheses to be tested are:

#### Hypothesis 1

The best EFA solution would be a three-factor one as evolved from the content validity study.

#### Hypothesis 2

The results of the EFA will be confirmed in a CFA in a different random subsample.

#### Hypothesis 3

Internal consistencies for the dimensions of the scale will show good reliability levels.

#### Hypothesis 4

The dimensions in the ACAR-PFS will show good convergent validity results with other scales measuring risk of maltreatment and protective factors against those risks.

## Results

### Descriptive Statistics

Descriptive statistics show that answers to items of the ACRAM-PFS all ranged from 0 to 2, and their average responses oscillate between 1.53 (SD = 0.75) of item 17, and 0.45 (SD = 0.72) of item 9. Items’ descriptive statistics are presented on Table 2, and inter-item correlations are displayed in Table 3. This table shows many statistically significant inter-item correlations, but the different effect sizes (from small correlations to large correlations), suggests that several different dimensions may underlie the responses to these items.

**Table 2:** Items’ descriptive statistics

Factor	Items	Mean ± *SD*
Children and adolescents’ resources	Item 1	0.69 ± 0.84
	Item 2	0.95 ± 0.89
	Item 3	0.89 ± 0.89
	Item 4	0.48 ± 0.76
	Item 5	0.78 ± 0.89
	Item 6	0.98 ± 0.94
Family/caregiver’s resources	Item 7	1.03 ± 0.88
	Item 8	1.01 ± 0.92
	Item 9	0.45 ± 0.72
	Item 10	0.60 ± 0.82
	Item 11	0.90 ± 0.87
	Item 12	1.03 ± 0.93
	Item 13	0.77 ± 0.88
Community resources	Item 14	0.78 ± 0.89
	Item 15	1.03 ± 0.93
	Item 16	1.18 ± 0.88
	Item 17	1.53 ± 0.75
	Item 18	0.96 ± 0.90

**Table 3:** Correlation coefficients among the items

	1	2	3	4	5	6	7	8	9	10	11	12	13	14	15	16	17
Item 1	–																
Item 2	.48**	–															
Item 3	.41**	.53**	–														
Item 4	.41**	.47**	.44**	–													
Item 5	.41**	.54**	.57**	.58**	–												
Item 6	.34**	.41**	.35**	.44**	.50**	–											
Item 7	.17**	.21**	.20**	.23**	.23**	.25**	–										
Item 8	.21**	.21**	.17**	.17**	.20**	.19**	.46**	–									
Item 9	.12**	.15**	.10*	.23**	.11**	.16**	.22**	.17**	–								
Item 10	.10*	.16**	.09	.19**	.15**	.17**	.34**	.30**	.48**	–							
Item 11	.18**	.17**	.17**	.19**	.17**	.15**	.37**	.28**	.37**	.47**	–						
Item 12	.14**	.22**	.17**	.12**	.14**	.12**	.32**	.31**	.46**	.46**	.47**	–					
Item 13	.13**	.23**	.21**	.16**	.17**	.21**	.39**	.46**	.47**	.47**	.46**	.64**	–				
Item 14	.38**	.38**	.34**	.35**	.32**	.34**	.30**	.22**	.30**	.31**	.36**	.23**	.34**	–			
Item 15	.27**	.33**	.21**	.31**	.28**	.28**	.20**	.13**	.08	.18**	.22*	.13**	.19**	.49**	–		
Item 16	.19**	.29**	.19**	.21**	.28**	.22**	.22**	.15**	.04	.18**	.27**	.18**	.22**	.33**	.61**	–	
Item 17	.05	.15**	.06	.10*	.12**	.11*	.17**	.16**	.15**	.10*	.24**	.24**	.23**	.16**	.16**	.28**	–
Item 18	.05	.14**	.02	.11*	.19**	.14**	.14*	.17**	.16**	.14*	.23**	.17**	.33**	.24**	.37**	.44**	.26**

** = *p* < .01; * = *p* < .05

### Exploratory Factor Analysis

Hypothesis 1 stated that the best EFA solution would be a three-factor one as evolved from the content validity study. In order to test this hypothesis, EFAs were estimated with all 18 items of the initial version of the ACRAM-PFS using subsample 1. We performed three EFAs: model solutions of one, two and three-factors. Fit indexes for these three solutions as well as fit comparison can be consulted in Table 4. All fit indexes clearly pointed the three-factor model as the best fit. Standardized factor loadings of the three-factor EFA are shown in Table 5. This last table clearly shows that factor 1 is composed of items 1 to 6, loadings of items 7 to 13 are large with the second factor, while the remain items (14–18) loaded into the third dimension. Only items 8, 14, 15 and 18 showed small to moderate cross-loadings into different factors.

**Table 4:** Goodness-of-fit indexes for the tested models on subsample 1

Models	χ^2^	df	*p*	RMSEA	90% CI	SRMR	CFI
One-factor	763.947	135	< .001	.122	.113–.130	.174	.792
Two factors	407.327	118	< .001	.088	.079–.098	.108	.904
Three factors	168.238	102	< .001	.045	.033–.057	.059	.978

**Table 5:** Rotated factor loadings from the three-factor solution

	Factor 1	Factor 2	Factor 3
Item 1	.85*	.01	− .10
Item 2	.78*	.17*	< .01
Item 3	.73*	.12	− .11
Item 4	.79*	− .04	.06
Item 5	.81*	− .01	.13
Item 6	.66*	.01	.05
Item 7	.18	.49*	.10
Item 8	.27*	.40*	.02
Item 9	.08	.76*	− .11
Item 10	− .04	.72*	.01
Item 11	− .01	.51*	.35*
Item 12	.02	.89*	− .01
Item 13	< − .01	.89*	.03
Item 14	.26*	.13	.50*
Item 15	.09	− .28*	.98*
Item 16	− .01	− .22	.99*
Item 17	− .09	.15	.49*
Item 18	− .31*	.01	.88*

*p < .05

### Confirmatory Factor Analysis

**Fig. 1:**
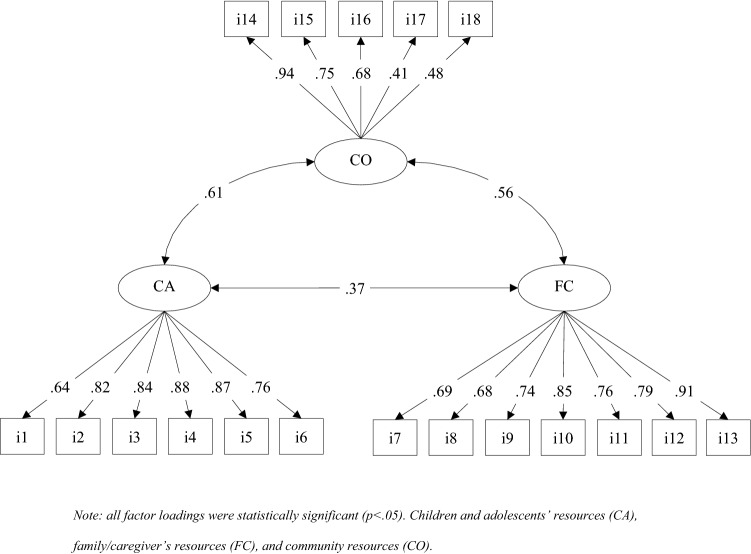
Standardized factor loadings of the CFA model in the overall sample. *Note*: all factor loadings were statistically significant (p < .05). *CA* Children and adolescents’ resources, *FC* family/caregiver’s resources, *CO* and community resources

Hypothesis 2 stated that the results of the EFA would be confirmed in a CFA in a different random subsample. Therefore, a CFA was used to test for this hypothesis. Having retained the three-factor EFA model, we used the second subsample to cross-validate the model, this time employing a CFA. Fit indexes showed adequate model fit to the data χ^2^ = 278.005, df = 132, p < 0.001, CFI = 0.955, SRMR = 0.084, RMSEA = 0.061, [90% CI: 0.051-0.071]. Standardized loadings are presented in Fig. 1. As in the CFA, all items loaded significantly into their theoretical factors, with the lowest standardized loading being 0.41. Figure 1 also shows that the dimensions were correlated, but these correlations were not as large as to jeopardize the discriminant validity of the factors.

### Internal Consistency

Hypothesis 3 stated that internal consistencies for the dimensions of the scale will show good reliability levels. To test this hypothesis reliability estimates for the dimensions of the scale were calculated. The scale demonstrated adequate internal consistency for the second subsample. Reliability estimates for the three dimensions can be considered very good. McDonald’s omega for each factor were: ω = 0.917 for the dimension of children and adolescent’s resources, ω = 0.914 for the dimension of family/caregiver’s resources and ω = 0.798 for the dimension of community resources.

### Convergent Validity

Hypothesis 4 states that the dimensions in the ACAR-PFS will show good convergent validity results with other scales measuring risk of maltreatment and protective factors against those risks. Accordingly, correlations of ACRAM-PFS’ dimensions with the CTQ-SF factors and the protective dimension of the C-CAPS were calculated. In general, correlations were statistically significant and in the expected direction. In general, the dimension of family caregiver’s resources is the one that has larger (negative) correlations, which is sensible, given that the CTQ-SF measures risks of abuse and maltreatment by the caregivers. Also important to note that the dimension of protective factors correlates highly with all dimensions of the ACRAM-PFS. All correlations estimated among the ACRAM-PFS dimensions were statistically significant. Correlations among the ACRAM-PFS and CTQ-SF and C-CAPS are included on Table 6.

**Table 6:** Correlations among the dimensions of the ACRAM-PFS and the CTQ-SF and C-CAPS dimensions

Measures’ dimensions		CTQ-SF					C-CAPS
		EN	PN	SA	PA	EA	PF
ACRAM-PFS	CA	− .23**	− .15**	.03	.07	.02	.21**
	FC	− .68**	− .55**	− .12**	− .15**	− .28**	.64**
	CO	− .29**	− .25**	− .04	.01	− .05	.30**

** = *p* < .01; * = *p* < .05. ACRAM-PFS: *CA* children and adolescents’ resources, *FC* family/caregiver’s resources, and *CO* community resources. CTQ-SF: *EA* emotional abuse, *PA* physical abuse, *SA* sexual abuse, *EN* emotional neglect, and *PN* physical neglect. C-CAPS: *PF* protective factors ()

## Discussion

This research aimed to develop a scale, the ACRAM-PFS, to assess protective factors for child maltreatment and offer evidence on its psychometric properties, given the need to have unbiased and psychometrically sound scales of these factors (Brumley et al., 2019; Gabrielli & Jackson, 2019; Kugler et al., 2019). The scale has been designed for its use in Spanish speaking countries. In particular, this study attempts to provide evidence on the structural validity, internal consistency and convergent validity of the scale.

Most assessment tools of child maltreatment have focused on the identification of risk factors (Ross & Vandivere, 2009). The development of ACRAM-PFS is based on the theoretical framework of strength-based assessment (Navarro et al., 2022). It postulates that assessing the presence or absence of protective factors completes the information provided by risk factors, pointing out the features of the case that should be exploited during the intervention (Ogg et al., 2010). In this respect, this scale was designed to identify the protective factors that decrease the likelihood of child maltreatment or reduce, moderate or compensate its negative consequences. Following the socio-ecological model proposed by Belsky (1980, 1993), indicators were designed in order to cover the protective factors related to three systems involved on child maltreatment: to children and adolescents’ resources, family/caregiver’s resources, and community resources.

We explored the factor structure in the subsample 1 using EFA and then replicated the best model solution in the subsample 2, employing CFA. Model fit indexes showed a very good fit of the structure to the data and all items loaded significantly on their corresponding dimensions. As it was expected, the three-factor solution was the one with the best fit indexes given the construction of the scale based on factors of the individual, the close environment and the distant systems postulated by Belsky (1980, 1993). This responds to the need to use a model that integrates information from different systems to allow practitioners and researchers to make more accurate and comprehensive assessments for the child maltreatment (Begle et al, 2010; Chen & Chan, 2016).

We estimated internal consistency using McDonald’s mega based on standardized factor loadings from the best fitting model. The results suggested that ACRAM-PFS displays an adequate internal consistency with all omegas higher than 0.70.

Regarding convergent validity, the ACRAM-PFS dimensions are all correlated in the expected direction. In general, the ACRAM-PFS dimensions presented significant negative correlation coefficients with CTQ-SF (Bernstein et al., 2003) and positive with C-CAPS protective factors dimension (Ezzo & Young, 2012). Regarding the CTQ-SF, dimensions of emotional and physical neglect, and are better related than emotional, sexual and physical abuse with all subscales from the ACRAM-PFS. The protective factors dimension of the C-CAPS showed statistically significant and positive correlations with the different dimensions of the ACRAM-PFS. The present results provide evidence of adequate criterion validity for the scale.

The results of this study show that ACRAM-PFS has optimal psychometric properties to assess protective factors from an ecological and strengths-based framework. As pointed out by previous studies (Gomez & Fliss, 2019; Meng et al., 2018), the assessment of protective factors is essential to contribute to early detection and to establish interventions based on the personal, family and contextual potentials of the child or adolescent. Following Shockley McCarthy et al. (2021), the findings of this study have a direct implication for professionals working with maltreated children, as the use of scientific tools ensures objectivity in diagnoses, supports decision-making and enables the translation of risks and strengths into intervention goals and strategies.

In short, the complex reality of child maltreatment requires comprehensive assessments that take into account both negative and positive aspects (Calheiros et al., 2021). Furthermore, given the pandemic caused by COVID-19, the attention and protection of children is particularly important, as they are known to be a particularly vulnerable population in the face of such disasters (Galea et al., 2005), and particularly in a context where social isolation and economic stress resulting from the pandemic may have exacerbated the risk of abuse (Lee et al., 2022; Self-Brown et al., 2022). Research on children's well-being in adverse situations emphasizes the importance of individual, family, and environmental resources in promoting positive development and outcomes in the face of disasters (Zhang et al., 2021a, 2021b).

This study shows the importance of assessing protective factors to promote resilience and enhance the autonomy and positive growth and development of children and adolescents in the face of adversity (Yule et al., 2019). In sum, turning risks into strengths and promoting the potential of maltreated children and adolescents is essential for the development of successful professional interventions to ensure their well-being. For this reason, the creation of a set of indicators (the ACRAM project which includes the ACRAM-PFS) that assesses both risk factors and protective factors is an advantage for child welfare workers in that they will be able to use a standardized measure to support decision-making. In addition, the use of a single set of indicators allows for a much better integration of information coming from different areas related to maltreatment and allows for more accurate communication between child welfare and legal workers. Research has demonstrated the superior predictive ability of protective factors on victim improvement after maltreatment intervention because these variables are more malleable than risk factors. In short, the creation of measures of protective factors is necessary as they will provide a way for child welfare workers to be able to demonstrate the improvement of their interventions (Ogg et al., 2010).

### Limitations

This study has both strengths and limitations. First, the statistical analyses were conducted on a sample composed by institutionalized youths selected with non-probabilistic methods from a region of Spain. This fact limited the generalization of the results to other contexts where legislation and the attendance related to child protective services could be different. In addition, Hartman et al. (2009), report gender differences in protective factors which, in this case, have not been reviewed. Another limitation of this study is that, in addition to gender differences, there may be other cultural differences that mediate the impact of protective factors on child maltreatment (Yu et al., 2022). On the other hand, this is the first scale in Spanish language to provide data of validity and internal consistency dealing with protective factors for child maltreatment, including variables related to the different systems that origin this kind of situations. In addition, this scale is integrated into a set of indicators that, together, provide the information needed to make accurate decisions and plan interventions in the best way.

## Conclusions

Given the large number of factors involved in child maltreatment situations and their heterogeneity, there is an unmet need to employ and implement measurement instruments that collect information in a rigorous and comprehensive way. The present research describes the development and provides evidence of the psychometric properties of the ACRAM-PFS, that is included in the ACRAM project. As far as we know, this is the first scale for the evaluation of protective factors based on the theoretical framework of the socioecological models. The results support that ACRAM-PFS is a reliable and valid measure for assessing protective factors against child maltreatment and can serve as a key tool for designing strengths-based intervention strategies tailored to the real needs of children and adolescents. This study consolidates of the ACRAM battery as the first to allow a precise and exhaustive analysis of child maltreatment.

## Appendix

See Table 7

**Table 7:** Translated version of the Adolescent and Children in Risk of Abuse and Maltreatment Protective Factors Scale (ACRAM-PFS)

Factor	Items
Children and adolescents’ resources	1. The child or adolescent has its own self-care resources and personal capacities to anticipate situations that may pose a personal or social risk (invitation from peers to commit a crime, attacking others, etc.)
	2. The child has empathic capacity (puts him/herself in the other person's place, understands his/her emotions, etc.)
	3. The child describes himself/herself coherently according to the reality around him/her (is aware of family risk, does not deny that situations of abuse and/or risk exist)
	4. The child manages his or her emotions appropriately
	5. The child adequately verbalizes what he/she is thinking and feeling
	6. The child is getting on according to his or her developmental stage
Family/caregiver’s resources	7. The child feels that there is one or more figures in the family environment who make him/her feel supported: listened to and understood, feels better when the figure is close, feels the figure is accessible and available, that he/she can trust him/her, etc
	8. There is contact with the biological family and this is positive for the social and emotional development of the child (in cases where the child is separated from his or her biological family)
	9. The primary caregiver has parenting and developmental skills, as well as the ability to anticipate and address family conflicts and propose alternatives
	10. The primary caregiver and/or the partner of one of them, with whom the child lives, have a mutually supportive relationship
	11. The family has social support and community networks
	12. The main caregiver shows a positive attitude towards interventions and professional action
	13. The primary caregiver understands and accommodates in a balanced way the needs of the child arising from his or her situation of vulnerability and separation from the family of origin
Community resources	14. The child has different relationship groups and/or peers with socially adjusted behaviour with whom he/she carries out leisure and/or community activities
	15. The child is integrated into the school and is a positive factor in the child's socialization
	16. There are educational staff with whom the child feels emotionally and affectively attached, as well as teachers who attend to the child's educational needs and deal with difficulties that may hinder his/her integration
	17. There is adequate coordination between the welfare administrations (health, education, social services and/or others) that collaborate with the family and the child
	18. An improvement in the emotional condition and sociability of the child is observed after diagnosis and medical treatment
